# Cytotoxic Flavonoids from the Leaves and Twigs of *Murraya tetramera*

**DOI:** 10.3390/molecules26051284

**Published:** 2021-02-26

**Authors:** Chun-Xue You, Kun Zhang, Xin Li, Jing Liu, Wen-Juan Zhang, Xiao-Xue Yu

**Affiliations:** 1 Tianjin Key Laboratory of Agricultural Animal Breeding and Healthy Husbandry, College of Animal Science and Veterinary Medicine, Tianjin Agricultural University, Tianjin 300384, China; youchunxue@mail.bnu.edu.cn (C.-X.Y.); 1903030108@stu.tjau.edu.cn (X.L.); 2003020131@stu.tjau.edu.cn (J.L.); 2 Department of Hepatopancreatobiliary Surgery, Tianjin Nankai Hospital, Tianjin 300100, China; zhangk08@lzu.cn; 3 Graduate School, Tianjin Medical University, Tianjin 300070, China

**Keywords:** *Murraya tetramera*, flavonoid, B16, MDA-MB-231, cytotoxicity

## Abstract

Cytotoxic flavonoids of *Murraya tetramera* were investigated in this study. A novel flavonoid and twelve known flavonoids, including seven flavones (**1**–**7**), three flavanones (**8**–**10**), and three chalcones (**11**–**13**) were isolated from the leaves and twigs of *Murraya tetramera*. Chemical structures were elucidated by NMR combined with MS spectral analysis, and the new compound (**6**) was confirmed as 3′,5′-dihydroxy-5,6,7,4′-tetramethoxyflavone. Furthermore, all the isolated flavonoids were evaluated for their cytotoxicities against murine melanoma cells (B16), and human breast cancer cells (MDA-MB-231) by CCK-8 assay. Among them, compounds **7**, **13**, and **5** exhibited potent cytotoxic activities against B16 cell lines (IC_50_ = 3.87, 7.00 and 8.66 μg/mL, respectively). Compounds **5**, **13**, and **12** displayed potent cytotoxicities against MDA-MB-231 cell lines (IC_50_ = 3.80, 5.95 and 7.89 μg/mL, respectively). According to the correlation of the structure and activity analysis, 5-hydroxyl and 8-methoxyl substituents of the flavone, 8-methoxyl substituent of the flavanone, and 3′,5′-methoxyl substituents of the chalcone could be critical factors of the high cytotoxicity. The results indicated that the active flavonoids have potential to be developed as leading compounds for treating cancers.

## 1. Introduction

Cancer is a leading cause of death among most countries in the 21st century, and the incidence and mortality are rapidly growing worldwide [1]. Melanoma of the skin among males and breast cancer among females are two of most prevalent cancers in 2019 [2]. Melanoma arises from epidermal melanocytes and induces 80% of the dermatological cancer-related deaths [3]. Breast cancer is the second major cause of cancer deaths in female worldwide [4]. Serious side effects and drug resistance caused by conventional cancer treatment of chemotherapy and radiotherapy remain the major problems during the treatment [5,6]. Accordingly, plant secondary metabolites have been attracting more attention in drug development owing to multiple factors, and the various compounds discovered in plants made them as a rich source of cancer drug candidates [7,8,9,10]. Thousands of flavonoids have been isolated from stems, flowers, fruits, roots, and barks of the plants, moreover, many effective cytotoxic flavonoids from various plants were considered as potential leading compounds for the development of anticancer drugs [8,11,12,13].

The genus *Murraya* (family Rutaceae) is a common plant source of polymethoxylated and polyhydroxylated flavonoids [14]. *Murraya tetramera* Huang (*M. tetramera*) is a small tree that is widely distributed in Guangxi and Yunnan provinces of China. The folk medicine has been applied for treating coughs, bronchitis, rheumatism, asthma, and traumatic injury, etc. [15,16]. *M. tetramera* contains various flavonoids, coumarins, alkaloids, and sesquiterpenes [17,18,19,20]. Some of the isolated compounds have exhibited significant cytotoxic effects [20,21,22].

To explore potent cytotoxic flavonoids from *M. tetramera* as potential leading compounds for treating cancers and make comprehensive utilization of its natural resources, a phytochemical investigation was carried out, and the cytotoxicities were evaluated against murine melanoma cells (B16) and human breast cancer cells (MDA-MB-231) by CCK-8 assay.

## 2. Results and Discussions

### 2.1. Flavonoids Isolated from M. tetramera

**Figure 1:**
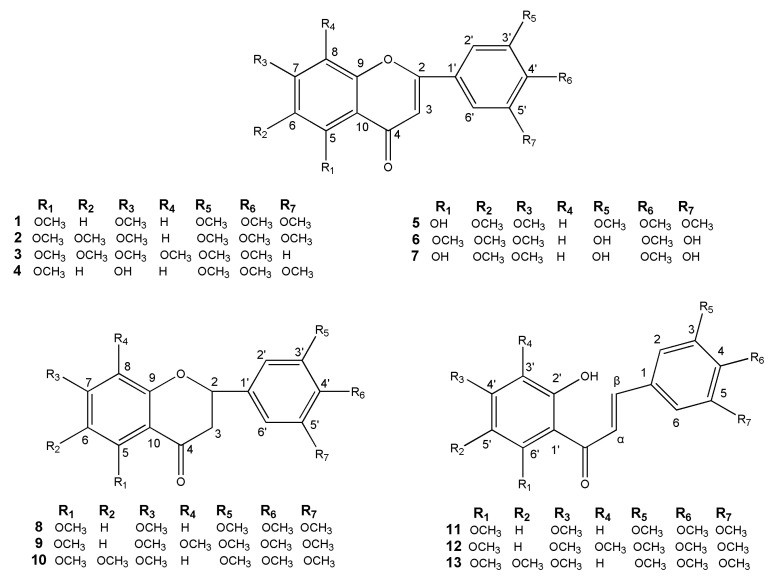
Chemical structures of flavonoids **1**–**13**.

A novel flavonoid and twelve known flavonoids, including seven flavones (**1**–**7**), three flavanones (**8**–**10**) and three chalcones (**11**–**13**) were isolated from the leaves and twigs of *M. tetramera*. The new one was identified as 3′,5′-dihydroxy-5,6,7,4′-tetramethoxyflavone (**6**) and the others were 3′,4′,5,5′,7-pentamethoxyflavone (**1**) [23], 5,6,7,3′,4′,5′-hexamethoxyflavone (**2**) [24,25], nobiletin (**3**) [26], 7-hydroxy-3′,4′,5,5′-tetra methoxyflavone (**4**) [27], 5-hydroxy-6,7,3′,4′,5′-pentamethoxyflavone (**5**) [28], 5,3′,5′-trihydroxy-6,7,4′-trimethoxyflavone (**7**) [29], 5,7,3′,4′,5′-pentamethoxyflavanone (**8**) [30], 3′,4′,5′,5,7,8-hexamethoxy flavanone (**9**) [31,32], 5,6,7,3′,4′,5′-hexamethoxyflavanone (**10**) [25], 2′-hydroxy-3,4,5,4′,6′-pentamethoxychalcone (**11**) [23,33], 2′-hydroxy-3,4,5,3′,4′,6′-hexamethoxychalcone (**12**) [33], and 2′-hydroxy-3,4,5,4′,5′,6′-hexamethoxychalcone (**13**) [34]. Their structures were shown in Figure 1. The ^1^H and ^13^C-NMR data of the twelve known flavonoids were listed in the Supplementary Materials.

### 2.2. Structure Elucidation of the New Flavone

**Figure 2:**
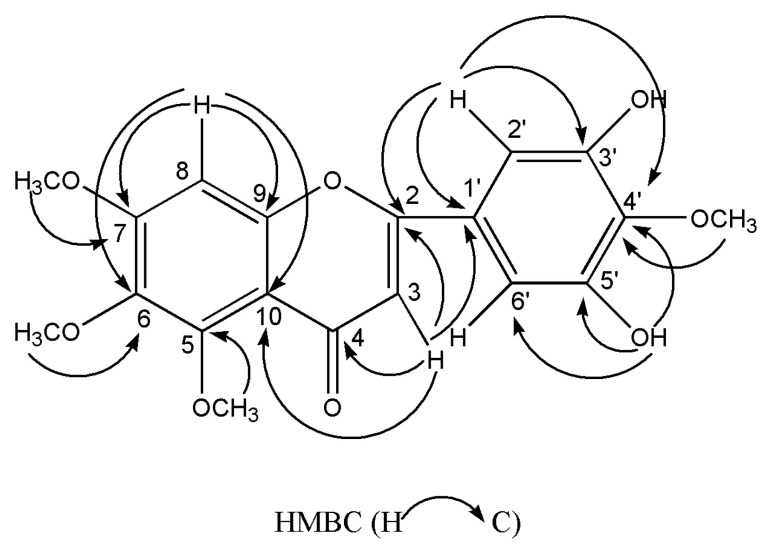
Key HMBC correlations of compound **6**.

Compound **6** was collected as yellow needles. The molecular formula of C_19_H_18_O_8_ was deduced from the peak at *m*/*z* 375.1075 [M+H]^+^ (calculated for C_19_H_19_O_8_, 375.1074) in the HR-ESI-MS and the 19 carbon resonances in the ^13^C-NMR data. The ^13^C-NMR exhibited the typical flavone signals at *δ*_C_ 176.0 (C-4), *δ*_C_ 161.0 (C-2) and *δ*_C_ 107.2 (C-3). The ^1^H-NMR displayed a typical flavone H-3 signal at *δ*_H_ 6.45 (1H, s), a characteristic flavone signal of H-2′ and 6′ at *δ*_H_ 7.00 (2H, s), and an aromatic proton at *δ*_H_ 7.10 (1H, s). Moreover, the ^1^H-NMR exhibited the existence of two hydroxy protons at *δ*_H_ 9.51 (2H, s), and four methoxyl peaks at *δ*_H_ 3.96, 3.80, 3.77 and 3.76 (each 3H, s). The HMBC displayed correlations arising from H-8 to C-10/C-6/C-9/C-7, from H-2′,6′ to C-1′/C-4′/C-3′,5′/C-2, from H-3 to C-10/C-1′/C-2/C-4, from 3′,5′-OH to C-2′,6′/, C-4′/C-3′,5′, from 7-OCH_3_, 5-OCH_3_, 6-OCH_3_ and 4′-OCH_3_ to C-7, C-5, C-6 and C-4′, respectively. The key HMBC correlations were indicated in Figure 2. Accordingly, compound **6** was deduced as 3′,5′-dihydroxy-5,6,7,4′-tetramethoxyflavone. The ^1^H and ^13^C-NMR data were listed in Table 1. All spectra are available in the Supplementary Materials.

### 2.3. Cytotoxicities of Isolated Flavonoids

Flavonoids **1**–**13** were evaluated for their cytotoxicities against B16 and MDA-MB-231 cell lines by CCK-8 assay and the results were displayed in Table 2. Among them, compounds **7**, **13**, and **5** exhibited potent cytotoxic activities against B16 cell lines (IC_50_ = 3.87, 7.00, and 8.66 μg/mL, respectively). Compounds **5**, **13**, and **12** displayed potent cytotoxicities against MDA-MB-231 cell lines (IC_50_ = 3.80, 5.95 and 7.89 μg/mL, respectively). However, flavonoids **1**, **6** and **11** showed weak anticancer efficacy against the two tested tumor cell lines (IC_50_ > 100 μg/mL).

The diverse cytotoxicities might be attributed to the different substituents of the flavonoids. Among flavones **1**–**7**, flavones **5**, **7**, and **3** exhibited higher cytotoxicities against B16 cells (IC_50_ = 8.66, 3.87, and 11.18 µg/mL) and MDA-MB-231 cells (IC_50_ = 3.80, 14.93, and 23.46 µg/mL) than others. Thus, 5-hydroxyl and 8-methoxyl substituents of the flavone were essential for high cytotoxicity, which corresponds to the state of literature [8,35]. In addition, compared with flavone **6**, flavone **2** showed a higher cytotoxicity against B16 and MDA-MB-231 cells with IC_50_ values of 14.74 and 34.19 µg/mL. Therefore, if the methoxy substituents in position 3′ and 4′ of flavone was substituted by hydroxyl substituents, as found in flavone **6**, the cytotoxicity was significantly reduced. Among the flavanones **8**–**10**, flavanone **9** exhibited the highest cytotoxicity against B16 and MDA-MB-231 cells (IC_50_ = 12.76 and 16.02 µg/mL). Thus, 8-methoxyl substituent of the flavanone could be a critical factor of the cytotoxic activity, which corresponds to the state of literature [35]. Among chalcones **11**–**13**, chalcones **12** and **13** exhibited higher cytotoxicities against B16 cells (IC_50_ = 11.53 and 7.00 µg/mL) and MDA-MB-231 cells (IC_50_ = 7.89 and 5.95 µg/mL) compared with chalcone **11**. Hence, 3′ and 5′-methoxyl substituents of the chalcone could be a major factor of the cytotoxic activity. Overall, according to the correlation of the structure and activity analysis, the position of methoxy and hydroxyl substituents in the flavonoids may be the major factors of the anticancer efficacy. Further investigation is essential to clarify the structure-active relationships.

## 3. Materials and Methods

### 3.1. General Information

The NMR spectrometer (Bruker Avance III, Bruker, Karlsruhe, Germany) was used to record the NMR spectra at 500 MHz (^1^H) and at 125 MHz (^13^C). The mass spectrometer (Bruker Q-TOF, Bruker, Karlsruhe, Germany) was used to measure the HR-ESI-MS. Preparative HPLC was carried out using a Rainbow Kromasil-C_18_ column (10 × 250 mm, 10 µm) on a Waters Delta Prep 4000 instrument with a dual λ absorbance detector (Waters 2487, Waters, Milford, USA). MCI GEL CHP20P of 75–150 μm (Kaiteki Company, Tokyo, Japan) was selected for column chromatography. Silica gel G plates were used for TLC analysis (Qingdao Haiyang Chemical Co., Ltd., Qingdao, China). The deuterated DMSO-*d*_6_ and CDCl_3_ were supplied by Cambridge Isotope Labo-ratories, Inc. (Andover, USA). DMEM, RPMI 1640 and fetal bovine serum were supplied by Gibco Inc. (New York, USA). Penicillin and streptomycin were provided by Solarbio science & technology Co., Ltd. (Beijing, China). CCK-8 reagent was obtained from Beyotime Biotechnology (Shanghai, China). All the analytical solvents of analytical grade were supplied by Beijing Chemical Plant (Beijing, China).

### 3.2. Plant Material

The leaves and twigs of *M. tetramera* were harvested at Xishuangbanna, Yunnan Province, China in May 2014 and were identified by Dr. Liu, Q.R. (College of Life Sciences, Beijing Normal University, Beijing, China). The certificate specimen (BNU-CMH-Dushushan-2014-05-025-001) was stored at the Herbarium of Faculty of Geographical Science, Beijing Normal University.

### 3.3. Extraction and Isolation

The methanol extract of leaves and twigs of *M. tetramera* was obtained from our previous study and 90 fractions were received from the methanol extract by eluting with a stepwise gradient of PE/EtOAc and CHCl_3_/CH_3_OH [36]. Fr. 55–57 (4.27 g), Fr. 59–60 (3.77 g), Fr. 66–67 (1.96 g), Fr. 68–69 (2.26 g) and Fr. 75 (1.51 g) were separated by MCI column chromatography with a mobile phase of EtOH-H_2_O (3:7, 5:5, 7:3 and EtOH), and then further purified by preparative HPLC using a stepwise gradient of MeOH-H_2_O (2:8→MeOH) to obtain flavone **1** (20 mg, 0.0008% yield), flavone **2** (150 mg, 0.006% yield), flavone **3** (60 mg, 0.0024% yield), flavone **4** (9.5 mg, 0.0004% yield), flavone **5** (2.1 mg, 0.00008% yield), flavone **6** (2.1 mg, 0.00008% yield), flavone **7** (2.8 mg, 0.0001% yield), flavanone **8** (20 mg, 0.0008% yield), flavanone **9** (200 mg, 0.008% yield), flavanone **10** (15 mg, 0.0006% yield), chalcone **11** (6.2 mg, 0.0002% yield), chalcone **12** (50 mg, 0.002% yield) and chalcone **13** (45 mg, 0.0018% yield), respectively. The compounds were stored at 4 °C in a refrigerator for subsequent experiments.

### 3.4. Cytotoxicity Assay

The cytotoxicities of flavonoids **1**–**13** were determined by the standard CCK-8 assay [20,37]. B16 (Number: GDC0039) were originally provided by China Center for Type Culture Collection (Wuhan, China) and MDA-MB-231 (Number: CL0208) were obtained from the Fenghui Biotechnology Co., Ltd. (Changsha, China). Doxorubicin hydrochloride (DOX), the positive control, was purchased from Dalian Meilun Biotechnology Co., Ltd. (Dalian, China). B16 cells were cultured in RPMI 1640 medium and MDA-MB-231 cells were cultured in DMEM medium. The medium supplemented with 10% fetal bovine serum (Gibco Inc.), 100 U/mL penicillin and 0.1 mg/mL streptomycin. The tested cell lines were incubated at 37 °C, 5% CO_2_ and 90% humidity in the CO_2_ incubator (Binder, Tuttlingen, Germany). Firstly, 100 μL of the cell suspension was seeded into each well of 96-well plates (6 × 10^3^ per well), and then incubated for 12–24 h to allow cellular attachment. After removing the medium, fresh medium containing seven concentrations of test compounds was added into cultured cells of 100 μL per well and incubated for 48 h. Secondly, 10 μL CCK-8 reagent was added into each well and placed in a CO_2_ incubator for 1 h. Finally, the absorbance was recorded using a microplate reader (Bio-Rad, Hercules, CA, USA) at 450 nm. The 50% inhibitory concentration (IC_50_) values were calculated using Probit analysis (SPSS V20.0).

## 4. Conclusions

A novel flavonoid and twelve known flavonoids, including seven flavones (**1**–**7**), three flavanones (**8**–**10**), and three chalcones (**11**–**13**) were isolated from the leaves and twigs of *M. tetramera*. The novel one (compound **6**) was identified as 3′,5′-dihydroxy-5,6,7,4′-tetramethoxyflavone. Results of cytotoxicity assay indicated that flavones **5** and **7** with 5-hydroxyl substituent, flavones **3** and flavanone **9** with 8-methoxyl substituent, chalcone **12** with 3′-methoxyl substituent and chalcone **13** with 5′-methoxyl substituent exhibited significant cytotoxic activities against B16 and MDA-MB-231 cell lines. According to the correlation of the structure and activity analysis, the position of methoxy and hydroxyl substituents in the flavonoids were the major factors of the high anticancer efficacy. The results indicated that the active flavonoids have potential to be developed as leading compounds for treating cancers. 

## Figures and Tables

**Table 1:** ^1^H and ^13^C-NMR data of flavone **6** in DMSO-*d*_6_.

Position	*δ*_H_ (ppm)	*δ*_C_ (ppm)
2		161.0
3	6.45, s	107.2
4		176.0
5		152.1
6		140.3
7		158.0
8	7.10, s	97.6
9		154.4
10		112.5
1′		126.4
2′	7.00, s	105.9
3′		151.6
4′		138.9
5′		151.6
6′	7.00, s	105.9
5-OCH_3_	3.80, s	62.3
6-OCH_3_	3.77, s	61.5
7-OCH_3_	3.96, s	56.9
4′-OCH_3_	3.76, s	60.3
3′, 5′-OH	9.51, s	

**Table 2:** Cytotoxicities of flavonoids **1**–**13** from *Murraya tetramera*.

Compound	IC_50_ ± SD (µg/mL)	
	B16	MDA-MB-231
1	>100	>100
2	14.74 ± 3.97	34.19 ± 3.38
3	11.18 ± 2.75	23.46 ± 2.95
4	14.97 ± 1.96	>100
5	8.66 ± 1.80	3.80 ± 1.49
6	>100	>100
7	3.87 ± 0.68	14.93 ± 2.71
8	13.03 ± 1.19	26.46 ± 2.53
9	12.76 ± 3.38	16.02 ± 1.12
10	23.55 ± 3.51	25.29 ± 3.84
11	>100	>100
12	11.53 ± 1.61	7.89 ± 1.71
13	7.00 ± 0.64	5.95 ± 0.65
DOX ^1^	0.51 ± 0.01	2.02 ± 0.65

^1^ Doxorubicin hydrochloride (positive control).

## Data Availability

All data supporting this study is available in the manuscript and the Supplementary Materials.

## Supplementary Materials

The following are available online, Figure S1: ^1^H-NMR spectrum of compound **6**, Figure S2: ^13^C-NMR spectrum of compound **6**, Figure S3: HMBC spectrum of compound **6**, Figure S4: HR-ESI-MS spectrum of compound **6**, Table S1: ^1^H-NMR data of the twelve known flavonoids, Table S2: ^13^C-NMR data of the twelve known flavonoids.

## Sample Availability

Samples of the compounds are available from the authors.
