# Supplementary material for: Cytotoxic Flavonoids from the Leaves and Twigs of Murraya tetramera
Source: Molecules. 2021 Feb 26;26(5):1284. doi: 10.3390/molecules26051284 (PMC7956623; doi:10.3390/molecules26051284)
Supplement: Supplementary file 1 [file molecules-26-01284-s001.pdf]

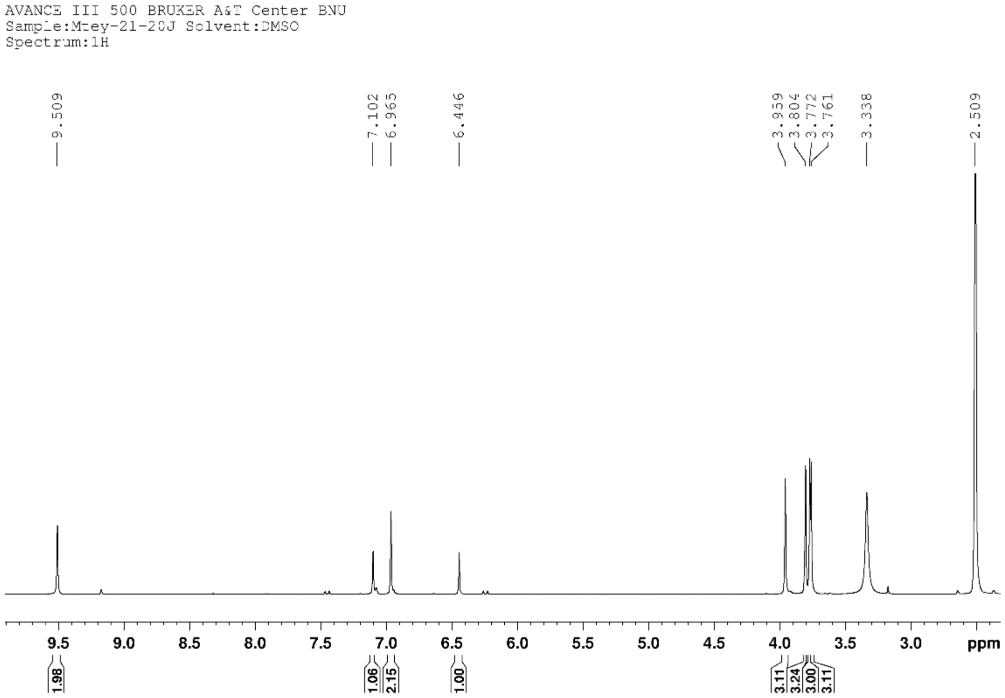

Figure S1: ^{1}H-NMR spectrum of compound **6**

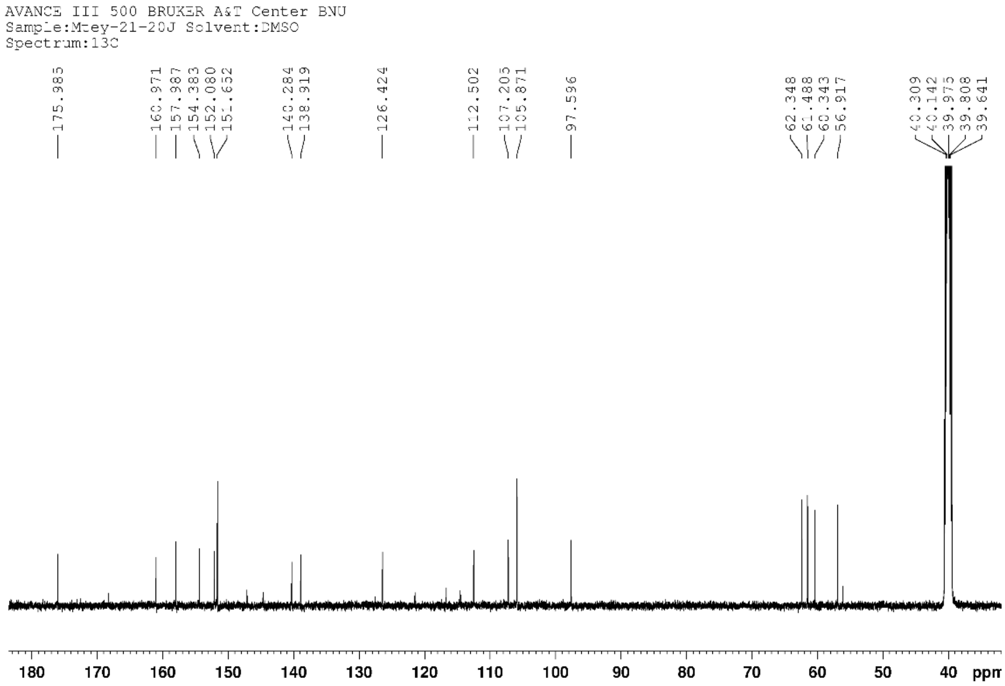

Figure S2: ^{13}C-NMR spectrum of compound **6**

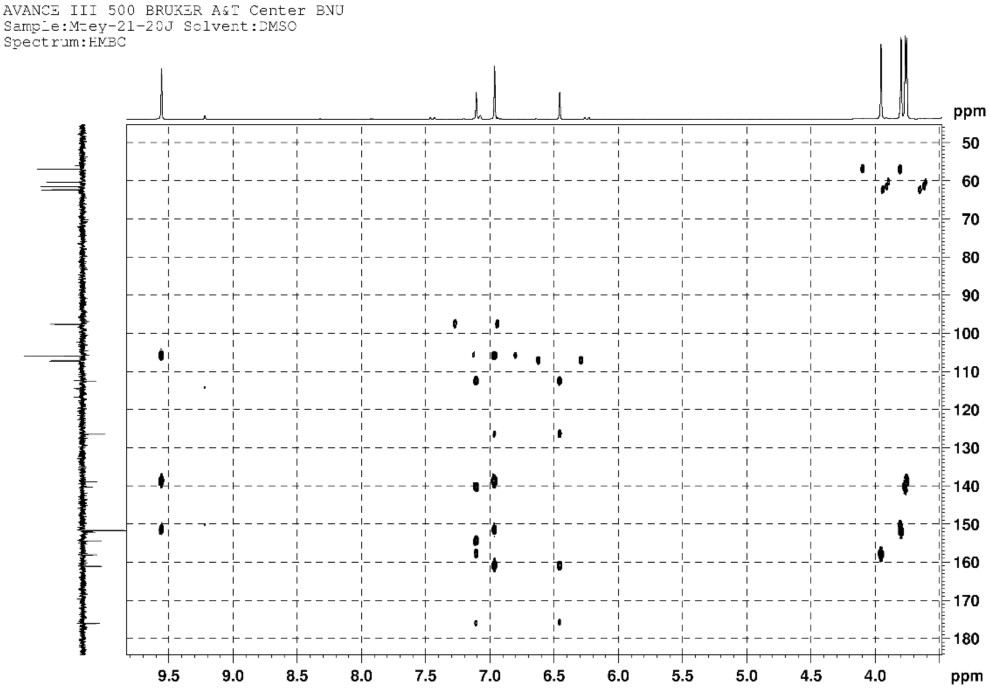

Figure S3: HMBC spectrum of compound **6**

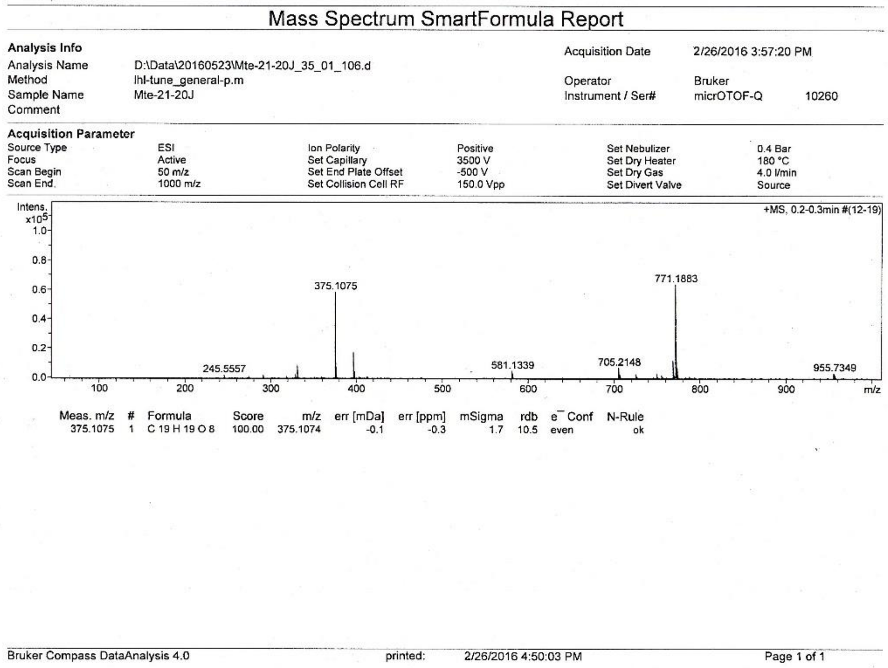

Figure S4: HR-ESI-MS spectrum of compound **6**

Table S1. ^{1}H-NMR data of the twelve known flavonoids.

Position	**1**[a]	**2**[a]	**3**[a]	**4**[b]	**5**[a]	**7**[b]	**8**[a]	**9**[a]	**10**[a]	**11**[a]	**12**[a]	**13**[a]
2							5.36, dd, *J*=13.5, 1.5 Hz	5.42, dd, *J*=12.0, 2.5 Hz	5.35, dd, *J*=13.5, 2.0 Hz	6.87, s	6.86, s	6.89, s
3	6.68, s	6.65, s	6.69, s	6.81, s,	6.64, s	6.68, s	H-a (3.05, dd, *J*=16.0, 13.5 Hz); H-b (2.81, dd, *J*=16.0, 1.5 Hz)	H-a(3.04, dd, *J*=17.0, 12.0 Hz); H-b(2.90, dd, *J*=17.0, 2.5 Hz)	H-a(3.04, dd, *J*=17.0, 13.5 Hz); H-b(2.79, dd, J=17.0, 2.0 Hz)			
6	6.41, d, *J*=1.5 Hz			6.39, s			6.20, s	6.17, s		6.87, s	6.86, s	6.89, s
8	6.59, d, *J*=1.5 Hz	6.83, s		6.62, s	6.59, s	6.86, s	6.13, s		6.40, s			
α										7.75, d, *J*=15.5 Hz	7.74, d, *J*=15.5 Hz	7.78, d, *J*=15.5 Hz
β										7.81, d, *J*=15.5 Hz	7.79, d, *J*=15.5 Hz	7.87, d, *J*=15.5 Hz
2′	7.09, s	7.09, s	7.43, d, *J*=1.5 Hz	7.27, s	7.11, s	7.02, s	6.70, s	6.72, s	6.70, s			
3′										6.15, d, *J*=2.0 Hz		6.33, s
5′			7.02, d, *J*=8.5 Hz							6.00, d, *J*=2.0 Hz	6.04, s	
6′	7.09, s	7.09, s	7.60, dd, *J*=8.5, 1.5 Hz	7.27, s	7.11, s	7.02, s	6.70, s	6.72, s	6.70, s			
-OCH3	3.98, s	4.02, s	4.13, s	3.90, s	4.01, s	3.95, s	3.93, s	3.98, s	3.97, s	3.94, s	3.98, s	3.96, s
-OCH3	3.97, s	4.01, s	4.05, s	3.90, s	4.00, s	3.77, s	3.92, s	3.96, s	3.92, s	3.94, s	3.97, s	3.95, s
-OCH3	3.97, s	3.98, s	4.00, s	3.81, s	4.00, s	3.74, s	3.92, s	3.90, s	3.92, s	3.94, s	3.94, s	3.95, s
-OCH3	3.95, s	3.98, s	3.99, s	3.74, s	3.96, s		3.89, s	3.90, s	3.91, s	3.92, s	3.94, s	3.93, s
-OCH3	3.94, s	3.94, s	3.97, s		3.96, s		3.86, s	3.88, s	3.89, s	3.87, s	3.92, s	3.93, s
-OCH3		3.94, s	3.97, s					3.85, s	3.86, s		3.87, s	3.86, s
5-OH					12.72, s	12.84, s						
7-OH				10.70, s								
2′-OH										14.33, s	13.97, s	13.71, s
3′, 5′-OH						9.57, s						

[a] Measured in $CDCl_3$. [b] Measured in DMSO-*d6*.

Table S2. ^{13}C-NMR data of the twelve known flavonoids.

Position	1[a]	2[a]	3[a]	4[b]	5[a]	7[b]	8[a]	9[a]	10[a]	11[a]	12[a]	13[a]
1										131.2	130.9	130.9
2	160.5	161.0	161.1	159.6	163.8	164.3	79.5	79.0	79.7	105.6	105.6	105.7
3	108.8	108.3	106.8	108.4	105.5	104.6	45.8	45.7	45.8	153.4	153.4	153.5
4	177.6	177.2	177.4	176.1	182.6	182.6	189.1	189.3	189.3	140.1	140.2	140.3
5	161.0	154.5	144.1	161.0	153.3	152.5	164.9	158.8	154.3	153.4	153.4	153.5
6	96.2	140.4	138.0	96.9	132.8	132.4	93.6	89.5	137.7	105.6	105.6	105.7
7	164.1	157.8	151.5	163.0	158.9	159.3	166.0	157.9	159.6			
8	92.9	96.3	148.4	95.9	90.7	91.9	93.3	131.0	96.4			
9	159.9	152.6	147.7	159.5	153.1	153.2	162.3	156.1	159.5			
10	109.2	112.9	114.8	107.7	106.2	105.7	106.0	106.3	109.2			
α										142.4	142.8	143.4
β										127.0	126.8	125.8
-C=O										192.4	193.0	192.7
1′	126.8	126.9	123.9	126.8	126.5	126.1	134.3	134.5	134.2	106.3	106.9	108.7
2′	103.4	103.4	108.4	104.0	103.8	106.4	103.2	103.1	103.3	166.2	159.4	162.7
3′	153.5	153.6	149.2	153.7	153.6	151.7	153.5	153.5	153.6	93.9	131.0	96.7
4′	140.9	140.9	151.9	140.5	141.5	139.5	138.2	138.0	138.3	168.4	158.4	160.2
5′	153.5	153.6	111.1	153.7	153.6	151.7	153.5	153.5	153.6	91.4	87.1	135.3
6′	103.4	103.4	119.7	104.0	103.8	106.4	103.2	103.1	103.3	162.4	158.5	154.9
$-OCH_3$	61.0	62.2	62.3	60.6	61.1	60.5	60.9	61.2	61.7	61.0	61.0	62.0
$-OCH_3$	56.5	61.6	62.0	56.7	60.9	60.4	56.2	60.9	61.4	56.2	60.8	61.3
$-OCH_3$	56.4	61.1	61.9	56.7	56.4	57.0	56.2	56.3	60.9	56.2	56.2	61.0
$-OCH_3$	56.4	56.4	61.8	56.3	56.4		56.2	56.2	56.2	55.8	56.2	56.2
$-OCH_3$	55.8	56.4	56.1		56.4		55.6	56.2	56.2	55.6	56.0	56.2
$-OCH_3$		56.4	56.0					56.1	56.2		56.0	56.1

[a] Measured in $CDCl_3$. [b] Measured in DMSO-*d6*.
